# Lactose Binding Induces Opposing Dynamics Changes in Human Galectins Revealed by NMR-Based Hydrogen–Deuterium Exchange

**DOI:** 10.3390/molecules22081357

**Published:** 2017-08-16

**Authors:** Chih-Ta Henry Chien, Meng-Ru Ho, Chung-Hung Lin, Shang-Te Danny Hsu

**Affiliations:** 1 Institute of Biological Chemistry, Academia Sinica, Taipei 11529, Taiwan; ctc40@cam.ac.uk (C.-T.H.C.); mho1@gate.sinica.edu.tw (M.-R.H.); chunhung@gate.sinica.edu.tw (C.-H.L.); 2 Department of Chemistry, National Taiwan University, Taipei 106, Taiwan; 3 Institute of Biochemical Sciences, National Taiwan University, Taipei 106, Taiwan

**Keywords:** galectins, protein dynamics, NMR spectroscopy, ligand binding

## Abstract

Galectins are β-galactoside-binding proteins implicated in a myriad of biological functions. Despite their highly conserved carbohydrate binding motifs with essentially identical structures, their affinities for lactose, a common galectin inhibitor, vary significantly. Here, we aimed to examine the molecular basis of differential lactose affinities amongst galectins using solution-based techniques. Consistent dissociation constants of lactose binding were derived from nuclear magnetic resonance (NMR) spectroscopy, intrinsic tryptophan fluorescence, isothermal titration calorimetry and bio-layer interferometry for human galectin-1 (hGal1), galectin-7 (hGal7), and the N-terminal and C-terminal domains of galectin-8 (hGal8^NTD^ and hGal8^CTD^, respectively). Furthermore, the dissociation rates of lactose binding were extracted from NMR lineshape analyses. Structural mapping of chemical shift perturbations revealed long-range perturbations upon lactose binding for hGal1 and hGal8^NTD^. We further demonstrated using the NMR-based hydrogen–deuterium exchange (HDX) that lactose binding increases the exchange rates of residues located on the opposite side of the ligand-binding pocket for hGal1 and hGal8^NTD^, indicative of allostery. Additionally, lactose binding induces significant stabilisation of hGal8^CTD^ across the entire domain. Our results suggested that lactose binding reduced the internal dynamics of hGal8^CTD^ on a very slow timescale (minutes and slower) at the expense of reduced binding affinity due to the unfavourable loss of conformational entropy.

## 1. Introduction

Galectins are a family of proteins that bind specifically to β-galactosides such as lactose, providing a way to recognise glycosylated proteins. Fifteen galectins have been discovered in mammals so far, and they can be found in the cytosol, nucleus, extracellular matrix, and circulation systems. On the basis of the carbohydrate recognition domain (CRD) arrangements, galectins are classified into three types [1,2]. Prototypical galectins are homodimers that consist of two identical CRDs. Galectin-1, -2, -5, -7, -10, -11, -13, -14 and -15 belong to this category. Tandem-repeat galectins contain two different CRDs that are linked by flexible linkers. These include galectin-4, -5, -8, -9, and -12. Galectin-3 (hGal3) is the only member of the chimera galectin family, which consists of one CRD and of a long non-lectin domain that is rich in proline, glycine, and tyrosine; the non-lectin domain is also responsible for the self-associate of hGal3. Despite the differences in the CRD arrangements, all the CRDs adopt the same β-sandwich fold consisting of two antiparallel β-sheets.

Galectins are implicated in a broad range of cellular functions, including apoptosis, immune response, and cell adhesion [3]. On the one hand, galectin-1 (hGal1) can induce the expression of anti-inflammatory cytokines such as IL-5, IL-10, and TGF-β in activated T cells [1]. On the other hand, it can inhibit the proinflammatory cytokines such as IL-2 [4], tumour necrosis factor-α (TNF-α) [5], and interferon-γ (IFN-γ) [6]; hGal1 is also involved in the T-cell apoptosis pathway by interacting with cell-surface glycoproteins such as CD7, CD29, and CD43 [7]. Galectin-7 (hGal7) plays an important role in corneal wound healing [8,9], the stimulation of aggressive lymphoma cells [10], while acting as a negative regulator of other cancer types of different histologies [11,12]. Unlike most galectins that are involved in immune responses and oncogenesis, galectin-8 (hGal8) is involved in cellular defence processes against bacterial infection and vacuolar damage [13]. Upon binding to damaged vacuolar, hGal8 recruits NDP52, an autophagy receptor, thereby inducing autophagosome formation to clear invaded bacteria [14,15].

As galectins are implicated in many important biological functions and diseases, recent years have witnessed emerging interests in the development of potent galectin inhibitors. Three lead compounds out of 30 lactoside and galactoside derivatives were found to block hGal1/HIV-1 interactions and reduce HIV-1 attachment to target cells [16]. A novel inhibitor that selectively binds to hGal7 with a dissociation constant (*K*_d_) of 140 μM was discovered from a phenyl thio-β-d-galactopyranoside library [17]. By far the most promising galectin inhibitor development has been of those that target hGal3. Screening for hGal3 inhibitors from a synthetic lacto-*N*-biose library has identified an *N*-naphthoyl derivative (*K*_d_ = 10.6 μM) with a good inhibitory effect [18]. A series of thiodigalactoside derivatives have been developed with much higher affinities [19,20]. Recently, a thiodigalactoside derivative with two triazole fluorophenyl extensions, known as TD139, exhibits a low nanomolar *K*_d_ against hGal3, and it is currently undergoing phase 1 study for the treatment of idiopathic pulmonary fibrosis [21,22].

**Figure 1:**
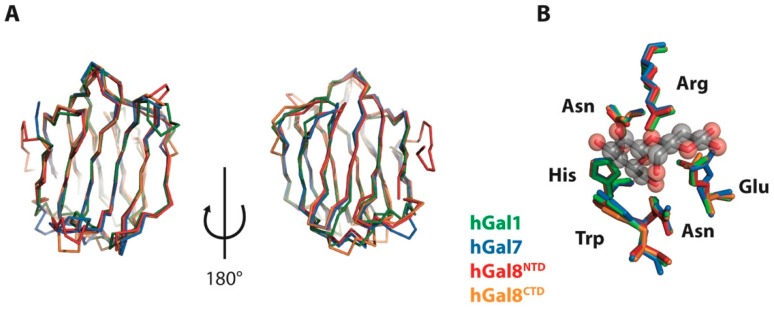
Superimposition of X-ray crystal structures of hGal1, hGal7, hGal8^NTD^, and hGal8^CTD^. (**A**) Ribbon representation of the crystal structures of hGal1 (protein database entry (PDB ID): 1W6N; green), hGal7 (PDB ID: 1BKZ; blue), hGal8^NTD^ (PDB ID: 3VKN; red), and hGal8^CTD^ (PDB ID: 3OJB; orange) are shown here on two sides; (**B**) Detailed views of structural alignment of lactose binding residues. The side-chains of individual residues are coloured in the same way.

The structural basis of substrate recognition is integral in inhibitor designs for better selectivity and affinity. Nonetheless, examination of the high-resolution structures of galectins shows almost identical backbone conformations (Figure 1). Limited conformational changes could be observed in the structures of galectins in complexes with different carbohydrate substrates. Indeed, it has been shown that the carbohydrate binding site of hGal3 has pre-organised water molecules with a carbohydrate-like framework of oxygen atoms, such that the displacement of these water molecules upon carbohydrate binding results in significant entropic gains by dehydration without the need for conformational changes on the protein side [23]. It, therefore, raises the question of why different galectins exhibit very different binding affinities for the same substrate. In the case of lactose, its *K*_d_ values for hGal1, hGal7 and the hGal8 N-terminal domain (hGal8^NTD^) and C-terminal domain (hGal8^CTD^) can vary by more than an order of magnitude without appreciable structural differences in the residues that are in direct contact with lactose, according to crystallographic analyses (Figure 1). A recent structural study indicated that the substrate binding preference may be associated with a salt bridge between the two ends of the loop on which a conserved arginine side-chain for carbohydrate binding is located [24], suggesting that structural and/or dynamics changes away from the direct contacting residues may play an important role in substrate recognition.

To further investigate the underlying mechanism by which galectins recognise their substrates, we employed nuclear magnetic resonance (NMR) spectroscopy and complementary solution state biophysical methods to obtain consistent *K*_d_ values for lactose binding to hGal1, hGal7, hGal8^NTD^ and hGal8^CTD^. Significant chemical shift perturbations (CSPs) in galectins were observed upon lactose binding. The impact of lactose binding on the dynamics of galectins was characterised by NMR-based hydrogen–deuterium exchange (HDX) analysis, which monitors changes in folding stability at a level of individual residues. Our results indicated that weaker lactose binding affinities in galectins are related to the increase in the internal folding stabilities, that is, restricted dynamics, upon lactose binding.

## 2. Results

### 2.1. Determination of Lactose Binding Affinity to Galectins

**Figure 2:**
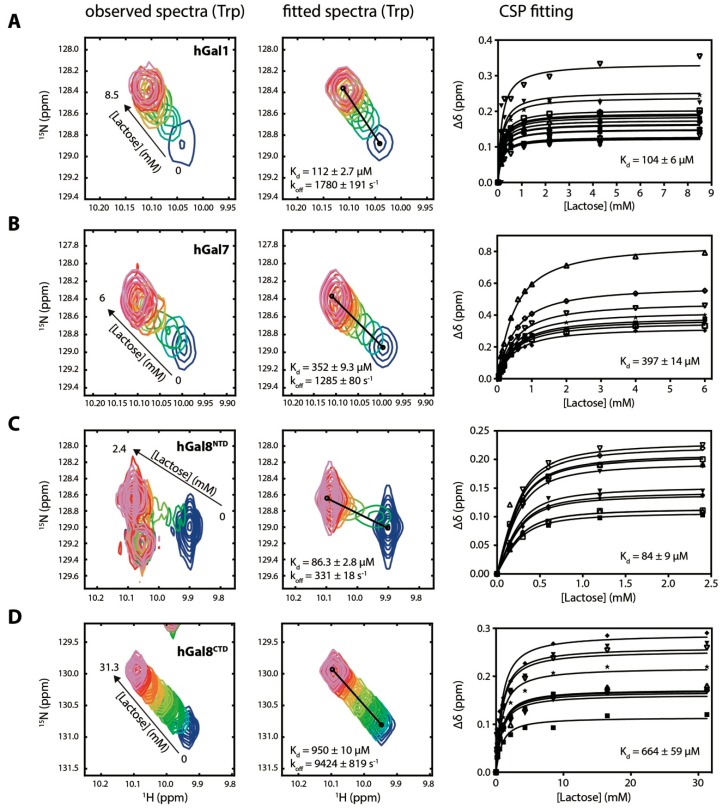
Lactose titration of galectins monitored by NMR spectroscopy. Observed and fitted ^15^N-^1^H band-selective optimised-flip-angle short-transient heteronuclear single-quantum correlation (SOFAST-HMQC) spectra corresponding to the tryptophan indole resonances of (**A**) hGal1, (**B**) hGal7, (**C**) hGal8^NTD^, and (**D**) hGal8^CTD^ are shown in the left and middle panels. The cross peaks are colour-ramped from blue to pink with increasing lactose concentration, as indicated by the titration points shown on the right panels with different final lactose concentrations as indicated by the arrows on the left panels. Details of the titration points are defined in Figure S1. Fitting results from TITAN program are indicated. Global fitting of the CSPs as a function of lactose concentration and the resulting *K*_d_ values for corresponding galectins are shown in the right panels. The titrations curves correspond to the residues that exhibit significant CSPs as defined in Figure 4, without severe overlaps throughout the titration to enable the calculations of individual CSPs at each titration point.

Quantitative analyses of ligand binding to different galectins have been reported using different biophysical techniques [25,26,27], but the *K*_d_ values for the same ligand binding can vary significantly depending on the analytical tools of choice. We, therefore, aimed to systematically compare the *K*_d_ values for lactose binding to different galectins using independent biophysical tools to alleviate bias resulting from detections based on different physico-chemical principles. We first carried out lactose titration into different galectins monitored by ^15^N-^1^H heteronuclear single-quantum correlation (HSQC) NMR spectroscopy. Similar studies have been reported for hGal1 [28] and hGal7 [27]. Using previously reported backbone NMR assignments of hGal1 [29] and hGal7 [30], and our recently reported backbone assignments of hGal8^CTD^ [31] and near-complete backbone assignments of hGal8^NTD^ (Chien and Hsu; unpublished data), we could monitor binding events at a residue-specific level. In particular, the indole amide of the strictly conserved tryptophan residues exhibited significant lactose-induced CSPs in the ^15^N-^1^H HSQC spectra, which serve as an excellent structural reporter for lactose binding (Figure 2, left panel). This was expected because the tryptophan side-chain is directly involved in lactose binding through CH–π interactions (Figure 1). In addition to CSPs, we also observed significant line broadening in some cases, which is associated with the timescale on which lactose binds to different galectins. In particular, hGal8^NTD^ exhibited significant line broadening in its indole resonance in the presence of intermediate lactose concentrations. We, therefore, performed a 2D NMR lineshape analysis using the program TITAN [32] to extract the *K*_d_ value and the off rate of lactose binding, *k*_off_, on the basis of a two-state binding model (Figure 2, middle panels; Figure S1). The lineshape analysis-derived *K*_d_ values for hGal1 and hGal7 were 112 ± 3 μM and 352 ± 9 μM, respectively; hGal8^NTD^ had the strongest lactose binding affinity with a *K*_d_ value of 86 ± 3 μM. In contrast, hGal8^CTD^ exhibited the weakest lactose binding with a *K*_d_ value of 950 ± 10 μM, which was about eightfold weaker than that of hGal8^NTD^. We also identified residues that exhibited CSPs greater than 2σ of the overall distributions and globally fit their CSPs to a single *K*_d_ value (Figure 2, right panels). The resulting *K*_d_ values were consistent with lineshape analysis-derived *K*_d_ values, except for hGal8^CTD^ (Figure 2, right panels; Table 1). This may have been ascribed to the significant dilution in the protein concentration during titration, which was necessary because of the need to titrate a large amount of lactose to saturate the system as a result of the weak binding affinity. According to the *k*_off_ values derived from the lineshape analysis, hGal8^CTD^ had the fastest off rate, which was 30 times faster than that of hGal8^NTD^, indicating a limited resident time of lactose in the binding pocket of hGal8^CTD^, and hence a weaker binding affinity (Table 2).

**Figure 3:**
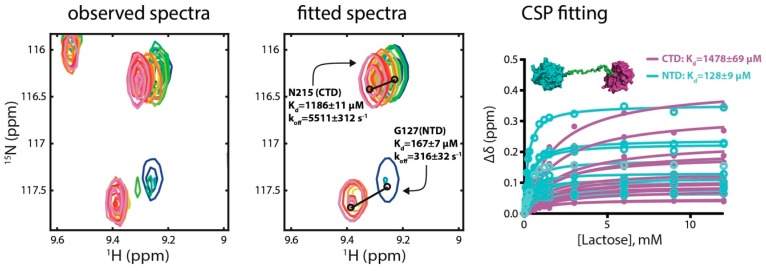
NMR titration of lactose binding to hGal8^full^ as a tandem-repeat construct. The expanded view of the 2D NMR spectra of hGal8^full^ is shown in the left panel, and ^15^N-^1^H cross peaks corresponding to G127 (NTD) and N215 (CTD) are colour-ramped from red to blue with increasing lactose concentration. The fitted spectra from the TITAN program are overlaid (in the same colouring scheme as the observed spectra) and shown in the middle panel. The lactose *K*_d_ derived from the TITAN fitting are indicated. The global fitting result of the CSPs as a function of lactose concentration is shown in the right panel. The titration curves of residues that corresponded to the NTD and CTD are coloured in cyan and magenta, respectively.

The advantage of using NMR titration to determine *K*_d_ values is the ability to simultaneously characterise microscopy binding events at different binding sites [33]. A full-length hGal8 (hGal8^full^) variant encompassing a 17-residue linker was used to examine whether lactose binding of the two CRDs would be affected by the presence of a flexible linker. By following the resonances that could be unambiguously assigned to the NTD and CTD, including the indole resonances, we globally fit the significant CSPs as a function of the lactose concentration and yielded two distinct *K*_d_ values, 128 ± 9 and 1478 ± 69 μM, corresponding to lactose binding to the NTD and to the CTD, respectively (Figure 3; Table 1). The same lineshape analysis was performed for hGal8^full^ titration experiments, which yielded similar *K*_d_ values to those derived from CSP data (Figure S2; Table 1). Although the *k*_off_ value of the NTD was consistent between hGal8^NTD^ and hGal8^full^, the *k*_off_ value of the CTD varied significantly; it was almost 2 times slower in the full-length construct (Table 2). The results indicated that the presence of domain crosstalk in hGal8^full^ resulted in reduced lactose binding affinities for both CRDs and decreased the lactose dissociation rate in the CTD.

The NMR-derived *K*_d_ values were cross-validated by three independent biophysical tools, namely, intrinsic fluorescence (Figure S3), bio-layer interferometry (BLI; Figure S4), and isothermal titration calorimetry (ITC; Figure S5), all of which showed consistent *K*_d_ values (Table 1). The strongest binding affinity towards lactose (84–93 μM) was found for hGal8^NTD^; the binding affinity was about 10-fold stronger than that of hGal8^CTD^ (664–950 μM), which had the weakest lactose binding affinity amongst the four galectin CRDs investigated herein, whereas hGal1 and hGal7 exhibited intermediate lactose *K*_d_ values of approximately 100 and 350 μM, respectively.

### 2.2. Structural Perturbations of Galectins upon Lactose Binding

**Figure 4:**
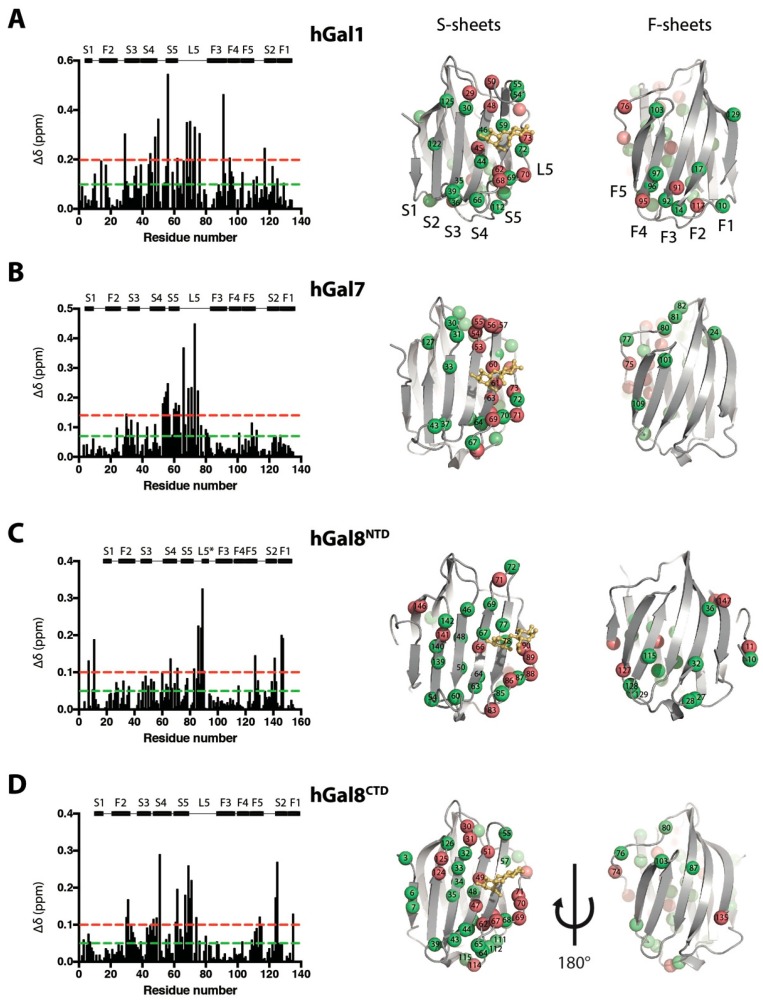
Structural mapping of lactose binding-induced CSPs in hGal1, hGal7, hGal8^NTD^, and hGal8^CTD^. Weighted CSPs upon lactose binding were plotted as a function of residue number (left panels) for (**A**) hGal1, (**B**) hGal7, (**C**) hGal8^NTD^, and (**D**) hGal8^CTD^. The values of 1σ and 2σ are shown by green and red dashed lines, respectively. The residues that exhibited CSPs greater than 1σ but less than 2σ (green spheres) and greater than 2σ (red spheres) are indicated on the corresponding crystal structures shown in the right panels. Crystal structure of hGal8^NTD^ shows a short β-sheet in L5 indicated by L5*.

While previous crystallographic analyses of lactose binding to different galectins showed no discernible structural rearrangements at either the backbone or side-chain levels [34,35], clear CSPs were observed by solution-state NMR spectroscopy. By mapping the significant CSPs onto the crystal structures of galectins CRDs, we found the highest CSPs to be located in close proximity to the lactose binding pockets for all galectin CRDs, including S4, S5, and L5 (Figure 4). Additionally, hGal8^NTD^ and hGal8^CTD^ showed significant CSPs in S2 and S3, possibly through perturbation of the β-sheet hydrogen-bond network. Long-range perturbations in F-sheets were observed for hGal1 and hGal8^NTD^, mainly at the bottom of F2, F3 and F4. In contrast, hGal7 only exhibited local perturbations upon lactose binding. The long-range structural perturbations of hGal1 and hGal8^NTD^ observed here were indicative of allostery, and were consistent with the previously reported allosteric perturbation upon lactose binding to hGal1 [28].

### 2.3. Impacts of Lactose Binding on the Dynamics of Galectins Monitored by NMR–HDX

Our NMR titration analyses revealed long-range CSPs in hGal1, hGal8^NTD^ and hGal8^CTD^ that were indicative of subtle structural and dynamic differences in these CRDs. To examine how lactose binding affects the folding dynamics of CRDs and to examine how dynamics is implicated in substrate recognition, we employed NMR–HDX analysis to probe the dynamics at the level of individual residues in the absence and presence of lactose [36,37]. The residue-specific HDX rates (*k*_HDX_) of hGal1, hGal7, hGal8^NTD^ and hGal8^CTD^ were obtained for most of the residues located in the secondary structure elements, that is, S-sheets and F-sheets (Figure S6). Many residues, however, including some within the secondary structure regions, exhibited rapid HDX such that their amide protons were fully exchanged beyond detection within the dead time of 5 min.

Overall, the residues that were located in the central β-strands of the β-sandwich, that is, S2, S3, S4, F2, F3 and F4, showed high protection factor (PF) values, with the exception of hGal7 exhibiting no protection in S4 (Figure S6). Significant differences in the PF values were observed in regions that were in close proximity to the carbohydrate binding site, especially in loop 5 (L5) that connected S5 and F3. For hGal1 and hGal8^NTD^, several loop residues in L5 showed high PF values, including E71, Q72, and R73 of hGal1, and K85, G87, E90, and D94 of hGal8^NTD^. For hGal7 and hGal8^CTD^, however, no appreciable HDX protection was observed in the same region, except for the weakly protected residues at positions 75 and 77 for hGal8^CTD^ (Figure S6D).

**Figure 5:**
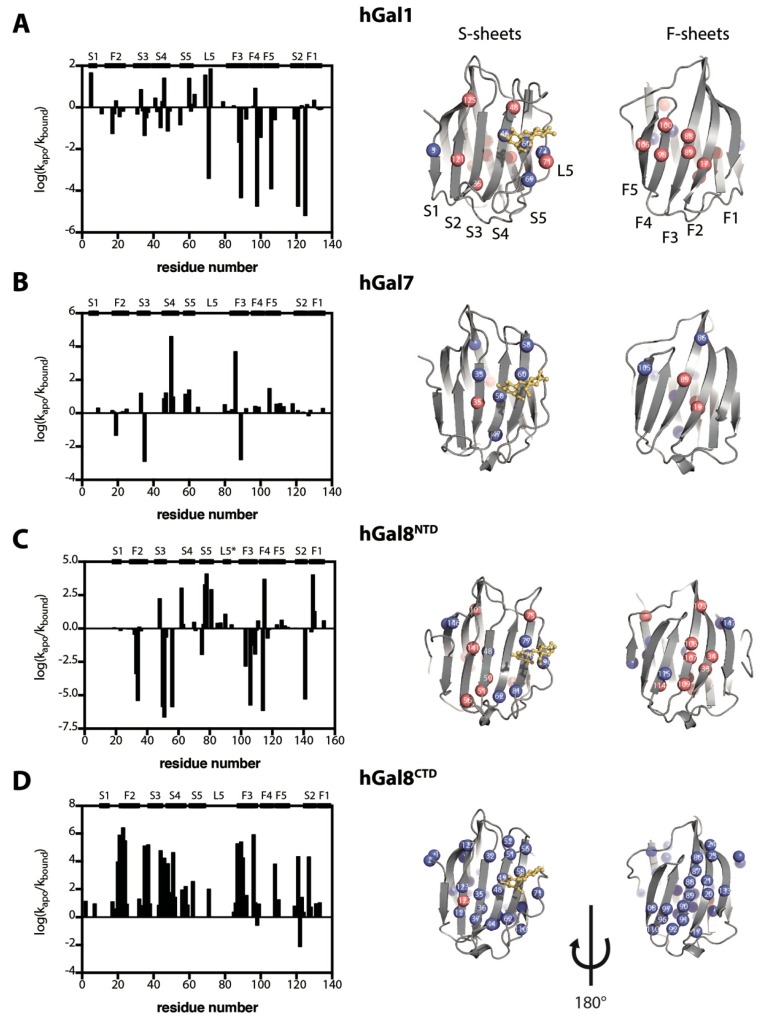
Comparison of galectin HDX rates between the apo- and lactose-bound forms. The logarithm of the ratio of the observed HDX rate for apo- and lactose-bound forms was plotted as a function of residue number for (**A**) hGal1, (**B**) hGal7, (**C**) hGal8^NTD^, and (**D**) hGal8^CTD^. The residues that exchanged over 10-fold faster (red sphere) and over 10-fold slower (blue sphere) in the bound form are indicated on the crystal structures of galectins (right panels). Crystal structure of hGal8^NTD^ shows a short β-sheet in L5 indicated by L5*.

We subsequently added saturating amounts of lactose and repeated the NMR–HDX analyses. The relative changes in the HDX rates were expressed in logarithm scales, log(*k*_apo_/*k*_bound_), as a function of the residue number (Figure 5, left panels). The residues that showed a significant difference (defined as the absolute log(*k*_apo_/*k*_bound_) value being larger than unity, i.e., |log(*k*_apo_/*k*_bound_)| > 1) were mapped onto the crystal structures of individual CRDs (Figure 5, right panels). The residues in close proximity to the carbohydrate binding pocket (S4, S5, and L5) showed significant increases in HDX protection for all galectins, as expected. Additionally, both hGal1 and hGal8^NTD^ showed a cluster of residues within the F-sheets (F2 and F3) with significant decreases in HDX protection, indicating the weakening of the hydrogen bonds within the F-sheets upon lactose binding, which took place on the opposite side of the CRD structures. Remarkably, the entire CRD structure of hGal8^CTD^ showed a significant reduction in HDX rates upon lactose binding (Figure 5D), indicating that lactose binding affects the global folding dynamics of hGal8^CTD^. The reduction in HDX rates, however, could be attributed to an enhanced hydrogen-bond stability or a decrease in solvent accessibility of the amide group of interest as a result of lactose binding, which should be a localised effect. Deconvoluting the two contributions is nontrivial. Nonetheless, one can assume that solvent sequestration upon lactose binding should be limited to residues in close proximity to the lactose binding site, that is, the S4–S5 strands and L5. This was indeed the case for hGal1, hGal7 and hGal8^NTD^, but was not so for hGal8^NTD^. Additional changes in the F-sheets (increased HDX rates in hGal1, hGal7 and hGal8^NTD^ and decreased HDX rates in hGal^CTD^) could be considered to be allosteric effects similar to the observations based on CSPs (Figure 4).

## 3. Discussion

### 3.1. Comparing K_d_ Values with Literature Values

In this work, we systematically compared the binding affinities for lactose binding to four human galectin CRDs and investigated the impacts of lactose binding on their structure and dynamics. We obtained self-consistent *K*_d_ values for lactose binding by using intrinsic fluorescence, BLI, ITC, and NMR CSP analyses, demonstrating the reliability of all these biophysical techniques in quantitative analyses of lectin–carbohydrate interactions without the need for exogenous fluorescence labelling (Table 1). We further carried out 2D NMR lineshape (TITAN) analyses to extract the dissociation rates associated with lactose binding, that is, the *k*_off_ values, in addition to the *K_d_* values that were consistent with the CSP-based fitting results and those derived from other biophysical techniques (Table 2).

Importantly, TITAN analyses revealed a markedly fast *k*_off_ value for hGal8^CTD^ (on the scale of 10^4^ s^−1^) that was 5–30 times faster than that of the other CRDs, suggesting the low lactose binding affinity of hGal8^CTD^ is due to the short resident time of its bound state. We further demonstrated the ability to simultaneously monitor lactose binding to the NTD and CTD of hGal8 as a tandem-repeat full-length hGal8 by 2D NMR spectroscopy (Figure 3). Two distinct *K*_d_ values were obtained from global fitting, and the stronger binding constant of hGal8^NTD^ (*K*_d_ ~ 130 μM) coincided with the apparent *K*_d_ value derived from surface plasmon resonance (SPR) analysis [38], while the 10-fold weaker binding constant of hGal8^CTD^ (*K*_d_ ~ 1500 μM) was associated with a very fast dissociation, *k*_off_ = 5511 s^−1^ (Table 2), that was too fast to be probed by SPR. Compared with the *K*_d_ and *k*_off_ values of isolated CRDs of hGal8, the presence of a flexible linker reduced the dissociation kinetics for both CRDs, but the respective binding affinities were also reduced, implying that the association rates, *k*_on_, for both CRDs were also reduced in the full-length hGal8, as *K*_d_ is defined as the ratio of *k*_off_ over *k*_on_.

While NMR spectroscopy affords exquisite atomic insights into how galectin CRDs bind to lactose, intrinsic fluorescence of the conserved tryptophan side-chain in galectin CRDs serves as a unique and sensitive structural probe for monitoring carbohydrate binding events. A robust singular value decomposition (SVD) analysis procedure was applied to deconvolute the spectral contributions of the apo- and lactose-bound forms to enable precise determination of the *K*_d_ values (Figure S3). Mayo and co-workers used NMR spectroscopy to obtain two lactose *K*_d_ values (50 μM and 250 μM) for hGal1 by fitting to a sequential binding model [28], while our intrinsic fluorescence data yielded an apparent *K*_d_ value of ca. 100 μM. Mayo and co-workers also showed that hGal7 exhibits two distinct lactose binding *K*_d_ values of 330 and 1100 μM [27], while we observe an apparent *K*_d_ value of 340 μM on the basis of intrinsic fluorescence. Using SPR, Ideo et al. showed that glutathione-*S*-transferase (GST)-fused hGal8^NTD^ exhibits a stronger lactose binding affinity (*K*_d_ = 80 μM) compared to GST-fused hGal8^CTD^ (*K*_d_ = 440 μM), while the measurement for a GST-fused tandem-repeat construct yielded an apparent *K*_d_ value of 140 μM, which was in between the values obtained from the two isolated CRDs [38]. In our case, we obtained a similar *K*_d_ value for hGal8^NTD^ (80 μM) and a much weaker *K*_d_ value for hGal8^CTD^ (800 μM), while the CRD-specific *K*_d_ values as a tandem-repeat construct could be derived from NMR-based titration, as discussed above.

### 3.2. Structural and Dynamics Impact on Galectins upon Lactose Binding

Structural mapping of the observed CSPs in hGal1 and hGal7 were consistent with the previously reported results [27,28]. Additionally, we observed long-range structural perturbations in both hGal8^NTD^ and hGal8^CTD^. The difference in lactose binding *K*_d_ values derived from isolated CRDs and tandem-repeat hGal8 suggested a possible inter-domain crosstalk between the two CRDs upon binding to lactose, manifested in reduced lactose binding kinetics, as discussed in the previous section. Despite the difference in their oligomeric state, we observed similar CSP patterns in hGal1 and hGal8^NTD^—pronounced CSPs in the F-sheets located on the opposite site of the lactose binding pocket—while most CSPs induced upon lactose binding were localised within the S-sheets of hGal7 and hGal8^CTD^ (Figure 4).

In contrast to the CSP analyses, NMR-HDX analyses revealed a different pattern for how lactose binding affects the global folding dynamics galectin CRDs. On the one hand, hGal8^CTD^ exhibited limited CSPs in the F-sheet, while it showed markedly reduced overall HDX rates in the lactose-bound form, particularly for many residues located in the F-sheet. On the other hand, a handful of residues in hGal1 and hGal8^NTD^ showed increased HDX rates both in the S-sheet and F-sheet (S4, S5, L5 and L8, in particular), and hGal7 exhibited the least difference in HDX between the apo- and lactose-bound forms (Figure 5). Lactose binding evidently induces opposing effects on the HDX characteristics amongst the four galectin CRDs studied herein. It is important to note however, that the effects of lactose binding on the observed HDX rates of individual CRDs were evaluated in relative terms without considering whether the amide group of interest was in the EX1 or EX2 regime. In other words, we focused on the relative changes in the observed HDX rates using the observed HDX rates of the apo forms as references, rather than the absolute values of the free energy of unfolding, the calculation of which requires the HDX to be in the EX2 regime [39]. Indeed, changes in the observed HDX rates reflected changes in the hydrogen-bond stability and/or solvent accessibility of the amide group of interest as a result of lactose binding; the latter should be a localised effect. Therefore, significant HDX rate changes that are distant from lactose binding sites are unlikely to be related to solvent sequestration due to lactose binding. Allosteric conformational and/or dynamics rearrangements are more likely to be responsible for the long-range effects. Considering that the association and dissociation of lactose are on a timescale of milliseconds or faster (Table 2), and that NMR-HDX analysis reports on a timescale of minutes or slower, we propose that the lactose binding affinity of galectins may be sensitive to conformational heterogeneity, that is, conformational entropy [40], and that lactose binding can also actively alter the solution structure ensemble interconverting on a much slower timescale than that of the binding kinetics.

Entropic contributions have been postulated to be an essential factor for substrate recognition in galectins on the basis of a comprehensive computational analysis of human galectins [41]. Our finding of an increasing in the F-sheet dynamics in hGal1 and hGal8^NTD^ upon lactose binding is consistent with the recent studies on hGal1, in which an increase in the backbone dynamics upon lactose binding (as reflected by increased backbone amide ^15^N order parameters and increased positional root-mean-squared fluctuations (RMSFs) of the backbone Cα atoms) was observed in the molecular dynamics simulations on a timescale of nanoseconds [28]. While order parameters and RMSFs are expressed on the nanosecond timescale, which is several orders faster than the timescale of lactose association/dissociation (milliseconds) and NMR–HDX (seconds and longer), our current experimental findings suggest that lactose binding may impact the dynamics of galectin CRDs over a broad range of timescales, from nanoseconds to minutes and beyond. Changes in dynamics across these timescales may all have contributed to the increase in conformational entropy required for the enhanced lactose affinities of hGal1 and hGal8^NTD^; likewise, it is plausible that the reduced conformational entropy due to the global reduction in HDX rates in hGal8^CTD^ may have been responsible for the poor lactose affinity. Given that galectin ligand binding processes are inherently dynamic [42], a better understanding of ligand binding dynamics will certainly help to improve ligand designs towards a higher affinity and specificity.

## 4. Materials and Methods

### 4.1. Preparation of Recombinant Galectins

The constructs encoding the open reading frames of hGal1, hGal7 and hGal8 with a hexahistidine tag at the N-termini were generated as described previously [24]. The sequence corresponding to hGal8^NTD^ was obtained by introducing a stop codon at position 156. The construct of hGal8^CTD^ was generated as described previously [31]. All recombinant CRDs were expressed in *Escherichia coli* BL21 (DE3) strain in LB media or in M9 minimal media for uniform ^13^C and/or ^15^N labelling. Over-expressed recombinant galectins were purified in phosphate buffer saline (PBS; 10 mM Na_2_HPO_4_ (pH 7.4), 137 mM NaCl, 1.8 mM KH_2_PO_4_, and 2.7 mM KCl) by nickel-nitrilotriacetic acid (Ni-NTA)-based affinity purification followed by size-exclusion chromatography to homogeneity (>95%) as judged by visual inspection of the corresponding Sodium dodecyl sulfate polyacrylamide gel electrophoresis (SDS-PAGE). The protein concentrations of individual samples were determined by optical absorbance at 280 nm using a UV-Vis spectrometer (V630, Jasco, Hachioji, Tokyo, Japan) together with the extinction coefficients at 280 nm of individual recombinant galectin variants, calculated by an online server ProtParam (http://web.expasy.org/protparam/) [43].

### 4.2. Intrinsic Fluorescence Spectroscopy

Concentrated lactose stock solution (300 mM) was prepared in PBS and was aliquoted by serial twofold dilution to generate a gradient of final lactose concentrations from 0 to 30 mM. The protein solution was mixed with the lactose solutions by using an electronic pipette (Eppendorf Multipette Xstream, Eppendorf, Hamburg, Germany) to reach a final protein concentration of 10 μM. The samples were subjected to intrinsic fluorescence measurements using a 3 mm quartz cell (Hellma) and a fluorimeter (Jasco FP-8500, Hachioji, Tokyo, Japan). All the experiments were performed at 298 K, and an excitation wavelength of 280 nm was used to collect the emitted fluorescence between 300 and 450 nm. The resulting titration data were processed by SVD analysis, as described previously [44,45].

### 4.3. Bio-Layer Interferometry

BLI experiments were carried out using an Octet Red96 system (FortéBio, Menlo Park, CA, USA), as described previously [33]. Briefly, super streptavidin (SSA) biosensors were used to immobilise recombinant galectins through biotinylation for the lactose binding experiments. Because all CRDs studied herein exhibited rapid lactose association and dissociation, BLI binding analyses were carried by fitting the steady-state response units as a function of the lactose concentration to extract the associated *K*_d_ values using Prism (GraphPad Software, San Diego, CA, USA).

### 4.4. NMR Titration Experiments

A series of ^15^N-^1^H HSQC spectra were collected using uniformly ^15^N-labelled galectins (0.3 mM) for the lactose titration experiments with 10–15 titration points, which corresponded to final lactose concentrations ranging from 0 to 30 mM. All NMR spectra were acquired at 298 K, using a Bruker AVANCE III 600 MHz spectrometer equipped with a cryogenic triple resonance probe (Bruker BioSpin, Rheinstetten, Germany), and the spectra were processed by Topspin (Bruker BioSpin, Rheinstetten, Germany) and NMRPipe [46] and analysed by SPARKY (Goddard and Kneller, SPARKY 3, San Francisco, CA, USA). Weighted CSPs of the backbone amide resonances were defined as

(1) $$\Delta \delta_{H,N}=\;\sqrt{{\left(\Delta \delta_{H}\right)}^{2}+0.13\times {\left(\Delta \delta_{N}\right)}^{2}}$$

where $\Delta \delta_{H}$ and $\Delta \delta_{N}$ are the observed chemical shift differences along the proton and nitrogen dimensions, respectively, with respect to the lactose-free ^15^N-^1^H HSQC spectrum. Significant CSPs, defined as those that were larger than two standard deviations, 2σ, were plotted as a function of the lactose concentration, and were globally fit to a single *K*_d_ value using the following function by Prism 6:

(2) $$[\mathrm{PL}]\;=\;\frac{\left(\left[P_{i}\right]+\left[L_{i}\right]+\left[K_{d}\right]\right)-\sqrt{{\left(\left[P_{i}\right]+\left[L_{i}\right]+\left[K_{d}\right]\right)}^{2}-4\left[P_{i}\right]\left[L_{i}\right]}}{2\left[P_{i}\right]}$$

where [PL] is the concentration of the complex formed, and [P_i_] and [L_i_] are the initial protein and ligand concentration, respectively.

HSQC spectra were also analysed by the TITAN program [32] using a two-state binding model. Protein and lactose concentrations as well as NMR acquisition parameters were provided as inputs for the data fitting. Eight peaks with significant CSPs were chosen to be fitted for each titration experiment. Error analysis was performed by the bootstrap resampling module in the program with 100 resampled spectra.

### 4.5. Isothermal Titration Calorimetry

ITC analyses of lactose binding to different galectin CRDs were conducted using a MicroCal VP-ITC instrument (Malvern Instruments Ltd., Malvern, Worcestershire, UK). The experiments were carried out at 298 K and in PBS. Lactose stock solution was prepared by dissolving lactose powder into PBS that was filtrated from protein samples to minimise buffer mismatch. The galectin sample (0.1 mM) was loaded into the sample cell and titrated with 10–40 mM lactose stock solution. The thermograms were extracted, processed and subjected to nonlinear regression using the built-in software based on Origin (OriginLab, Northampton, MA, USA) to determine the respective binding constants.

### 4.6. NMR Hydrogen–Deuterium Exchange

Uniformly ^15^N-labelled protein solution (0.5 mL of 0.3 mM) was lyophilised prior to the NMR-HDX experiments; 0.5 mL of 99.9% D_2_O was added to resuspend the lyophilised protein sample. The sample was immediately transferred into a 5 mm NMR tube to record a series of ^15^N-^1^H SOFAST-HMQC spectra [47] with a time interval of 10 minutes per spectrum for the first 20 spectra. Additional spectra with different time intervals from hours to days were recorded until the amide protons were fully exchanged into deuterons, that is, until no NMR signals were detected. The rate of HDX, *k*_HDX_, was extracted by fitting the observed cross-peak intensity as a function of the HDX time to a single exponential decaying function using the built-in relaxation module within Sparky. The protection factor was calculated using the following equation:

(3) $$\mathrm{PF}\;=\;\frac{k_{c}}{k_{\mathrm{HDX}}}$$

where PF is the protection factor and *k*_c_ is the intrinsic exchange rate of individual residues.

Galectin samples with saturating amounts of lactose were used to assess the impacts of lactose binding on the HDX processes of galectin variants. The additional protection against HDX as a result of lactose binding was calculated using the following equation:

(4) $${\mathrm{PF}}_{\mathrm{Lactose}}=\frac{{\mathrm{PF}}_{\mathrm{bound}}}{{\mathrm{PF}}_{\mathrm{apo}}}=\frac{k_{\mathrm{HDX},\;\mathrm{apo}}}{k_{\mathrm{HDX},\;\mathrm{bound}}}$$

## Figures and Tables

**Table 1:** Lactose *K*_d_ of hGal1, hGal7, hGal8^NTD^, hGal8^CTD^, NTD of full-length galectin-8 (hGal8^full^(NTD)) and CTD of full-length galectin-8 (hGal8^full^(CTD)) determined by four independent biophysical tools expressed in μM.

	NMR (CSP)	NMR (TITAN)	Intrinsic Fluorescence	BLI	ITC
hGal1	104 ± 6	112 ± 1	98 ± 8	110 ± 38	209 ± 13
hGal7	397 ± 14	352 ± 9	331 ± 6	347 ± 180	400 ± 2
hGal8^NTD^	84 ± 9	86 ± 3	88 ± 2	89 ± 26	93 ± 1
hGal8^CTD^	664 ± 59	950 ± 10	772 ± 56	873 ± 526	893 ± 25
hGal8^full^(NTD)	128 ± 9	167 ± 7	—	—	—
hGal8^full^(CTD)	1478 ± 69	1186 ± 11	—	—	—

**Table 2:** Lactose dissociation rate (*k*_off_) of hGal1, hGal7, hGal8^NTD^ and hGal8^CTD^ determined by lineshape analysis of 2D ^15^N-^1^H HSQC spectra.

	*k*_off_ (s^−1^)
hGal1	1780 ± 191
hGal7	1285 ± 80
hGal8^NTD^	331 ± 18
hGal8^CTD^	9424 ± 819
hGal8^full^(NTD)	316 ± 32
hGal8^full^(CTD)	5511 ± 312

## Supplementary Materials

Supplementary Materials are available online. Figure S1: Results of 2D NMR lineshape analysis for lactose titration of galectin CRDs; Figure S2: Results of 2D NMR lineshape analysis for lactose titration of hGal8^full^; Figure S3: Lactose titration of galectins monitored by intrinsic fluorescence spectroscopy; Figure S4: Lactose titration of galectins monitored by BLI; Figure S5: Lactose titration of galectins monitored by ITC; Figure S6: Topological mapping of protection factors of galectins in apo-form.
