# Supplementary material for: Lactose Binding Induces Opposing Dynamics Changes in Human Galectins Revealed by NMR-Based Hydrogen–Deuterium Exchange
Source: Molecules. 2017 Aug 16;22(8):1357. doi: 10.3390/molecules22081357 (PMC6152064; doi:10.3390/molecules22081357)
Supplement: Supplementary file 1 [file molecules-22-01357-s001.pdf]

Supporting information

Lactose Binding Induces Opposing Dynamics Changes in Human Galectins Revealed by NMR-Based Hydrogen-Deuterium Exchange

Chih-Ta Henry Chien [1,2], Meng-Ru Ho [1], Chung-Hung Lin [1, 2,3] and Shang-Te Danny Hsu [1,3,*]

[1] Institute of Biological Chemistry, Academia Sinica, Taipei 11529, Taiwan; ctc40@cam.ac.uk (C.-T.H.C.); mho1@gate.sincia.edu.tw (M.-R.H); chunhung@gate.sinica.edu.tw (C.-H.L.)
[2] Department of Chemistry, National Taiwan University, Taipei 106, Taiwan
[3] Institute of Biochemical Sciences, National Taiwan University, Taipei 106, Taiwan
* Correspondence: sthsu@gate.sincia.edu.tw; Tel.: +886-2-27889759

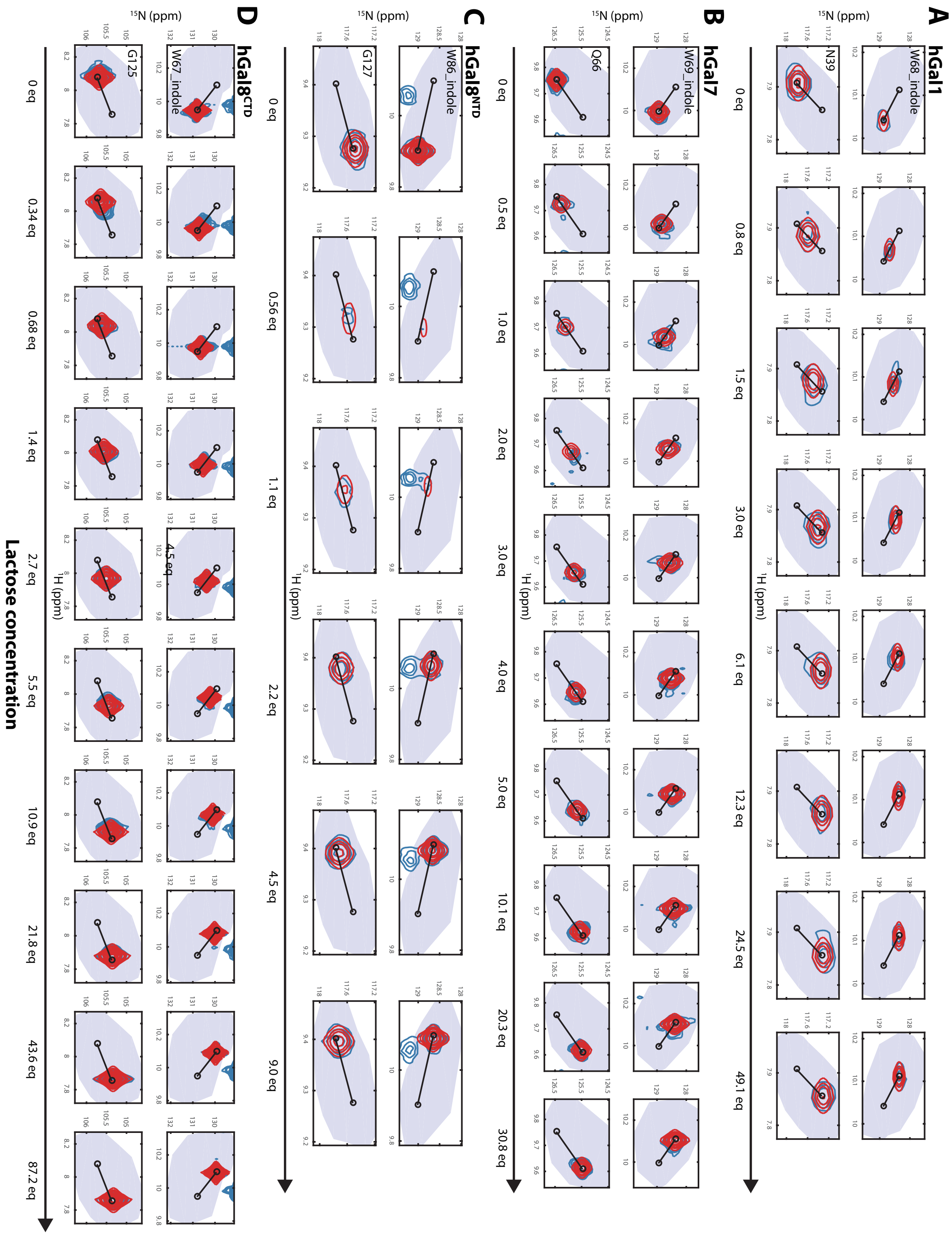

Figure S1. Results of 2D lineshape analysis for lactose titration of (A) hGal1, (B) hGal7, (C) hGal8^{NTD} and (D) hGal8^{CTD} using TITAN program. Observed peaks show in blue and fitted peaks show in red. Lactose molar ratio used in each experiment are indicated.

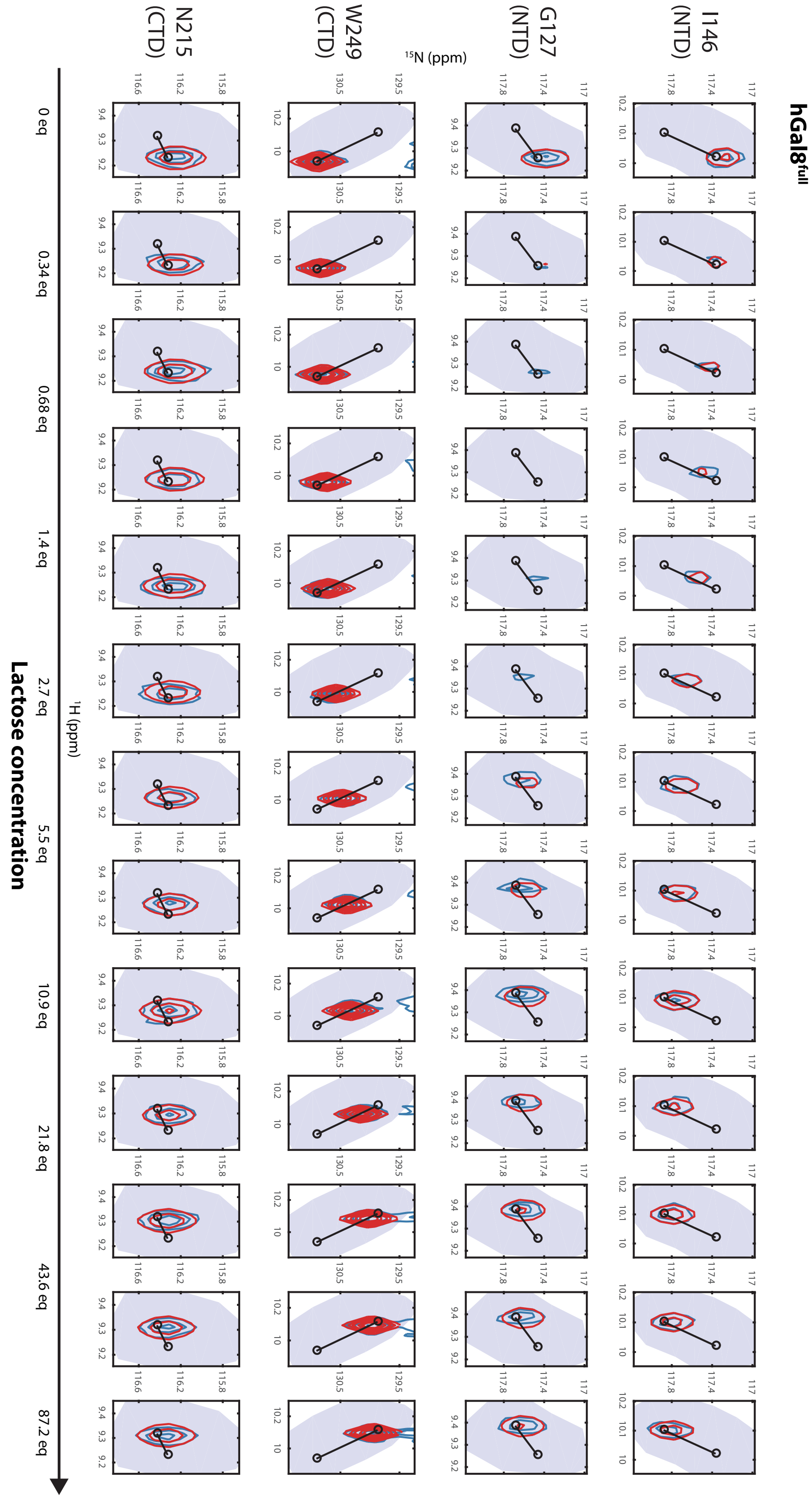

Figure S2. Results of 2D lineshape analysis for lactose titration of hGal8[full] using TITAN program. Observed peaks show in blue and fitted peaks show in red. Lactose molar ratio used in each experiment are indicated.

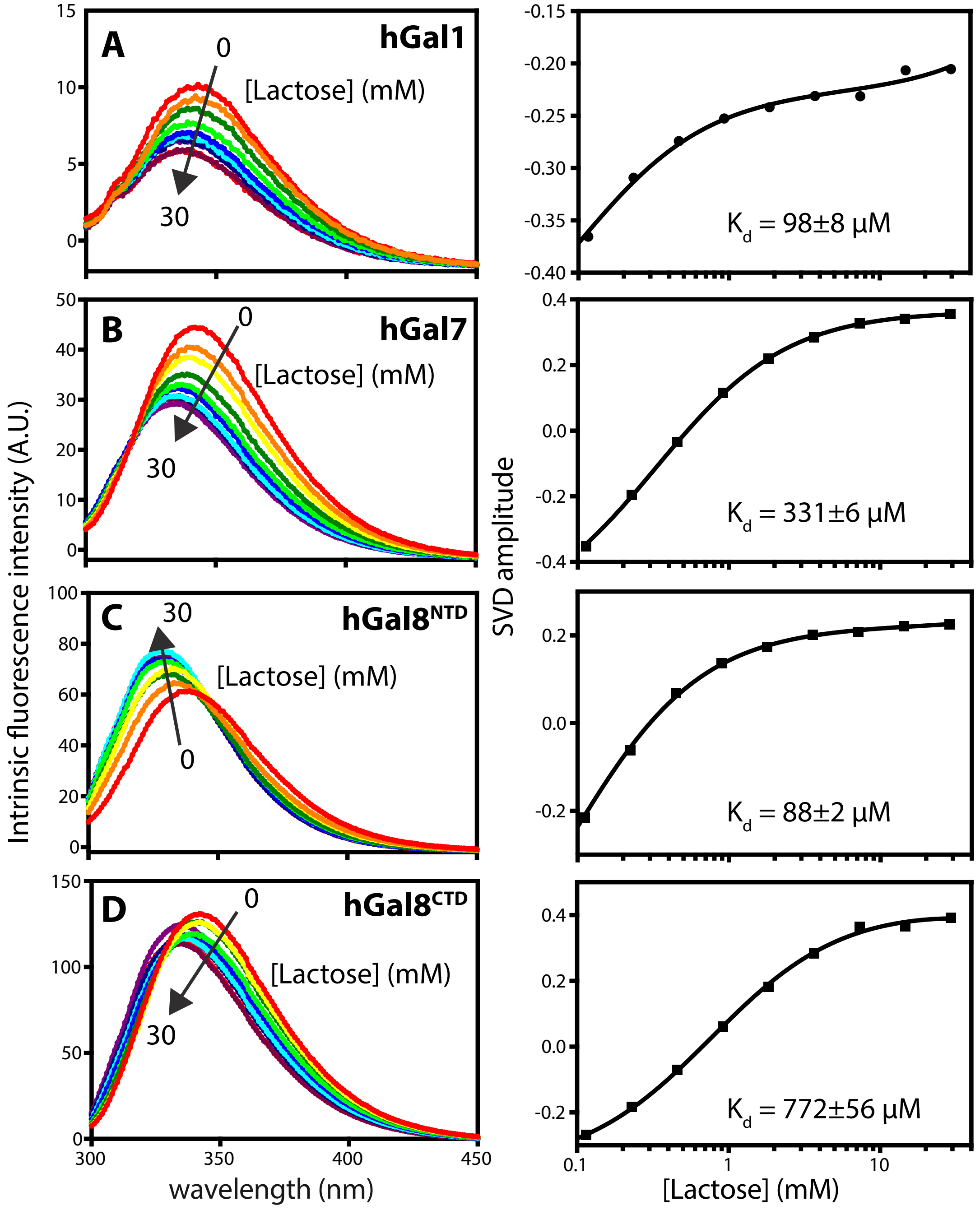

Figure S3. Lactose titration of galectins monitored by intrinsic fluorescence spectroscopy. Intrinsic fluorescence was used to determine the binding affinity of (A) hGal1, (B) hGal7, (C) hGal8^{NTD}, and (D) hGal8^{CTD}. Raw data are shown on the left panels, while the analysis and the K_d value show on the right panels.

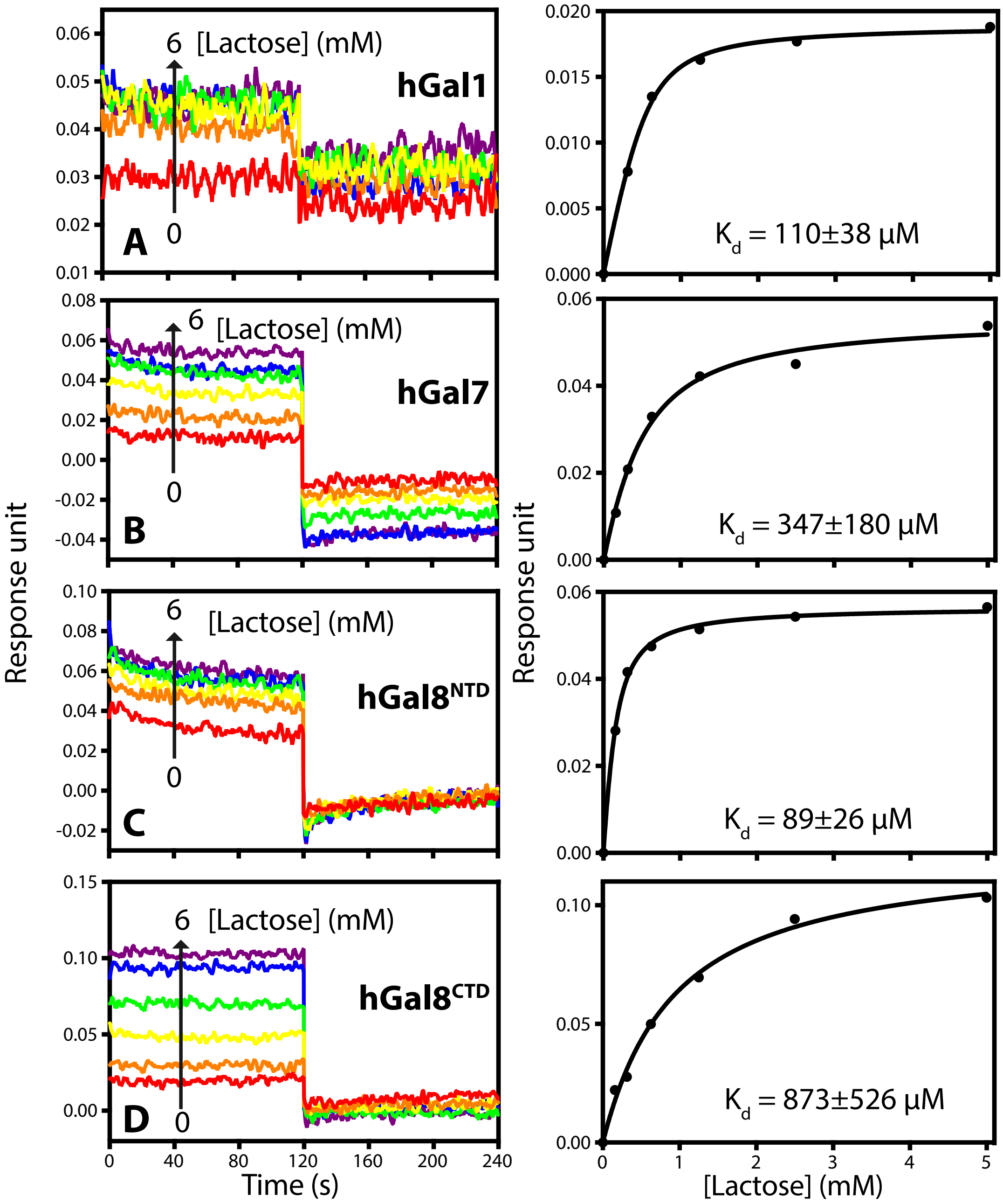

Figure S4. Lactose titration of galectins monitored by bio-layer interferometry. Bio-layer interferometry was used to determine the binding affinity of (A) hGal1, (B) hGal7, (C) $hGal8^{NTD}$, and (D) $hGal8^{CTD}$. Raw data are shown on the left panels, while the analysis and the K_d value show on the right panels.

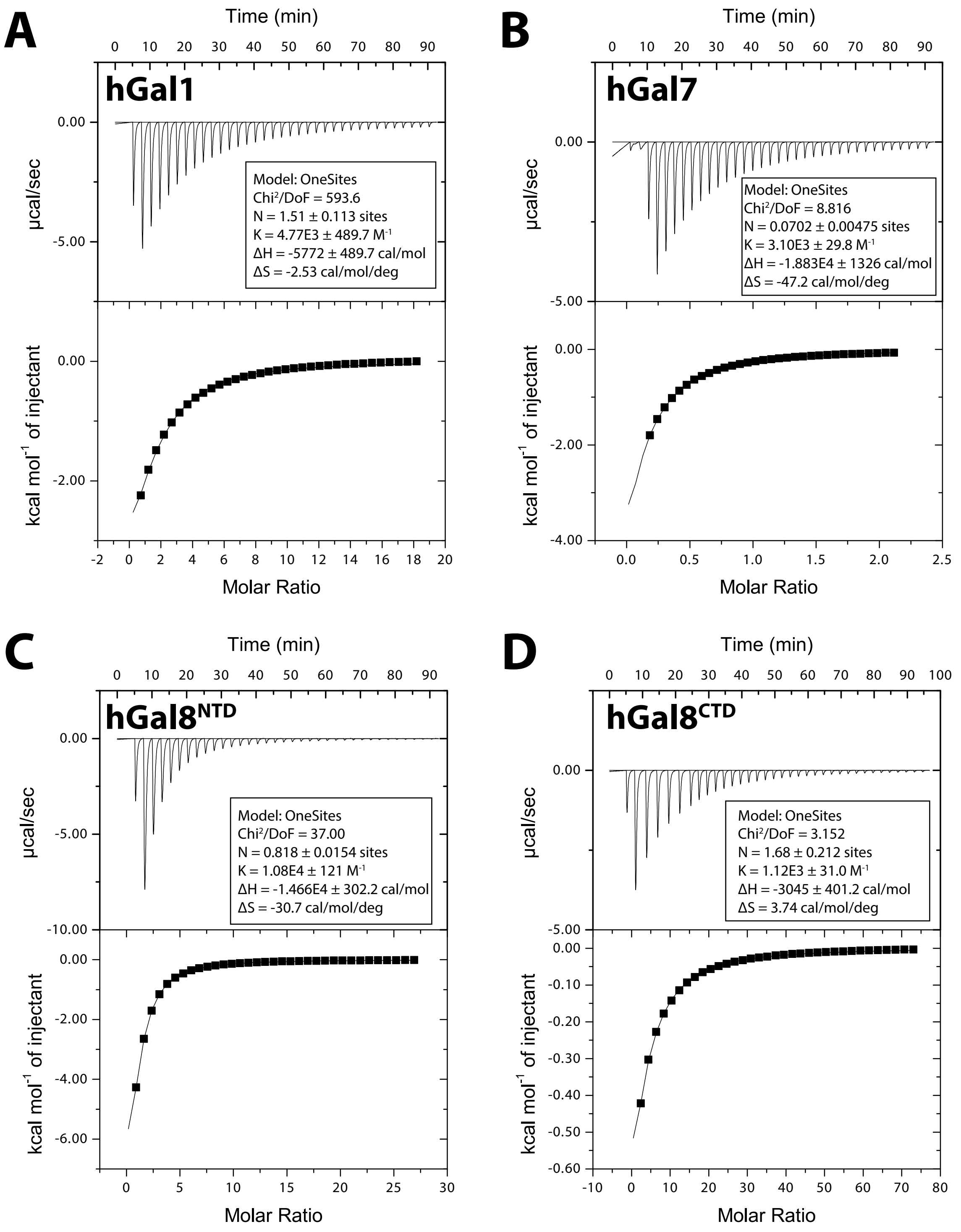

Figure S5. Lactose titration of galectins monitored by ITC. Lactose titration probed by ITC was used to determine the binding affinity of (A) hGal1, (B) hGal7, (C) hGal8^{NTD}, and (D) hGal8^{CTD}. Raw data are shown on the top panels, and fitting of the isotherms are shown on the bottom panels.

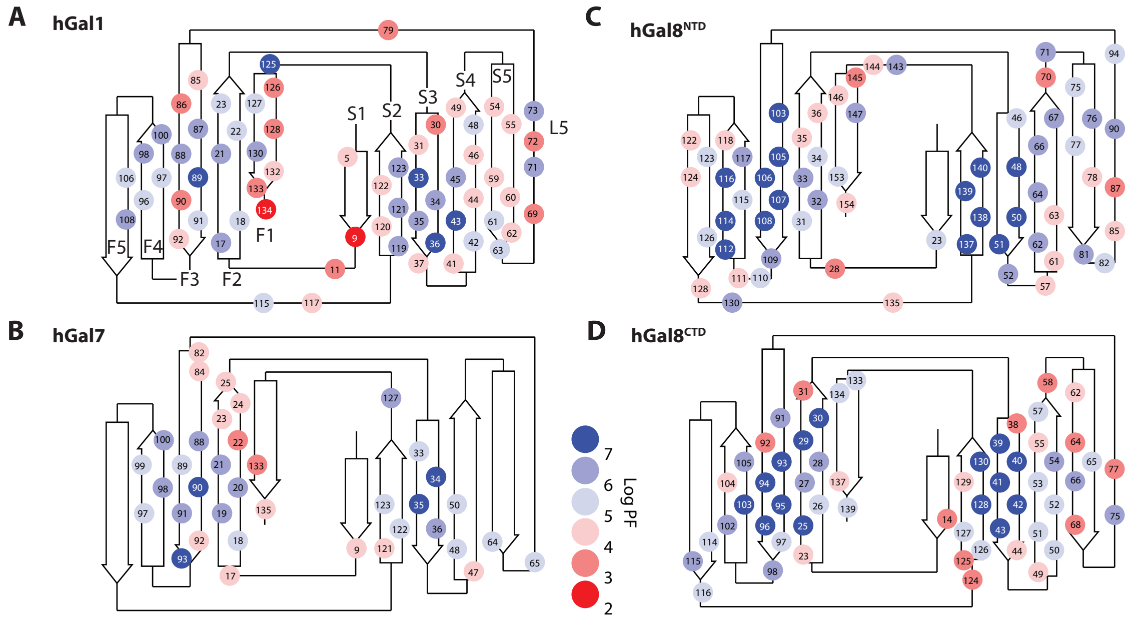

Figure S6. Topological mapping of protection factors of galectins in apo-form. Protection factors of the apo-forms of (A) hGal1, (B) hGal7, (C) hGal8^{NTD}, and (D) hGal8^{CTD} were mapped onto their and labeled with their respective residue numbers. Those residues that exchange too fast to be detected are not shown on the topology.
